# Bibliometric mapping of diabetes mellitus and sarcopenia research: hotspots and emerging trends

**DOI:** 10.3389/fmed.2025.1586308

**Published:** 2025-05-27

**Authors:** Min Wang, Qiyang Zhou, Xinyu Guo, Xiaolong Cui, Zhongquan Tang, Ting Ou, Xiaomin Zhao, Chen Liang, Peiyu Xu, Xinyu Dai, Jingqing Yao, Guozhong Ji, Yuntao Li

**Affiliations:** ^1^Department of General Practice, The Second Affiliated Hospital of Nanjing Medical University, Nanjing, China; ^2^Department of Neurology, The Second Affiliated Hospital of Nanjing Medical University, Nanjing, China

**Keywords:** diabetes mellitus, sarcopenia, pathological mechanisms, muscle function, bibliometric analysis, research trends

## Abstract

**Background:**

Diabetes mellitus and sarcopenia are chronic metabolic disorders characterized by bidirectional interactions, frequently coexisting as comorbidities whose interrelationship has garnered increasing scientific attention. This study pioneers a bibliometric analysis to systematically investigate their association, aiming to map the knowledge structure, evolutionary trajectories, current foci, and emerging frontiers within this area.

**Methods:**

We retrieved 2,773 publications from the Web of Science Core Collection from inception until December 26, 2024, and visual analyses were conducted using CiteSpace, VOSviewer, R, and Microsoft Excel. The analysis characterized disciplinary distributions, publication outputs, national/regional contributions, institutional collaborations, authorship networks, journal profiles, references, and keywords.

**Results:**

Annual publications demonstrated sustained growth, with the United States dominating scholarly contributions. Research exhibited marked interdisciplinary integration, although investigations linking type 1 diabetes mellitus with sarcopenia remain limited. Current research hotspots included shared pathological mechanisms such as insulin resistance and chronic inflammation, clinical characterization of specific subtypes such as sarcopenic obesity, imaging-based assessment of muscle dysfunction in diabetes, and the therapeutic efficacy of exercise as an intervention. Mechanistic exploration was determined to be the primary driver of domain advancement.

**Conclusion:**

The field has evolved from theoretical frameworks to clinical applications, highlighting the importance of uncovering common pathophysiological mechanisms and pinpointing potential therapeutic targets. Future priorities include refining screening and diagnostic protocols, optimizing preventive strategies, and developing personalized interventions. Cross-disciplinary innovations integrating multi-omics and precision medicine are poised to reshape this research landscape.

## 1 Introduction

Diabetes mellitus represents a multifactorial chronic disease predominantly marked by elevated blood glucose levels (1, 2), among which type 2 diabetes mellitus (T2DM) constitutes the most prevalent subtype. Its pathogenesis, involving intricate interactions among genetic, environmental, and lifestyle factors, remains incompletely understood. As reported by the International Diabetes Federation (2021), around 537 million individuals globally were affected by diabetes, with this number anticipated to increase to 783 million by 2045 (3). Another study predicts that global diabetes patients may reach 1.31 billion by 2050 (4). The escalating prevalence, particularly among older adults, has led to complex adverse effects across multiple organ systems and substantially elevated the risk of complications and mortality (2). As such, diabetes now represents a major global health concern in the 21st century. Beyond traditional complications such as microvascular and macrovascular diseases, diabetes patients often experience a range of emerging complications. Notably, sarcopenia is one of these complications (5), which is primarily driven by diabetes-induced mechanisms such as insulin resistance, chronic low-grade inflammation, oxidative stress, and dysregulated muscle protein metabolism (6, 7).

Sarcopenia is a progressive, systemic disorder of skeletal muscle, characterized by the rapid decline in muscle mass and function (8, 9). It significantly increases risks of falls, fractures, frailty, functional decline, and premature mortality. While diagnostic criteria vary across international organizations, common elements include reduced muscle mass, diminished muscle strength, and impaired physical performance (10). Globally, sarcopenia affects approximately 10–40% of individuals (11), with this variation influenced by population characteristics and assessment criteria. Among individuals aged 80 and older, the prevalence can reach 11–50% (12).

Notably, the prevalence of sarcopenia is markedly higher in individuals with diabetes compared to the general population (13). Previous studies reported an 18% prevalence of sarcopenia in diabetic patients (5), while a 2024 observational study from 60th Annual Meeting of the European Association for the Study of Diabetes found a prevalence of 38.30% in older adults with T2DM. Diabetes is now recognized as an independent risk factor for sarcopenia, and the onset and progression of sarcopenia significantly impact the prognosis of diabetes. In fact, there is considerable overlap in the pathophysiological mechanisms of both conditions (14). Diabetes and sarcopenia often coexist, and the coexistence of these conditions synergistically worsens metabolic dysregulation, elevates cardiovascular and all-cause mortality risks, and imposes substantial economic burdens on healthcare systems. Therefore, exploring the relationship between diabetes and sarcopenia is of critical importance.

Bibliometrics is a field that utilizes mathematical and statistical techniques to analyze literature both quantitatively and qualitatively (15, 16). It seeks to delineate the mechanisms of scholarly communication, patterns of disciplinary evolution, and distribution-citation dynamics within scientific literature. Through systematic quantification, it serves as a powerful tool for mapping emerging frontiers and intellectual hotspots across specialized research domains (17, 18).

While existing research has examined the link between diabetes and sarcopenia, a systematic bibliometric analysis remains lacking. This study aims to fill this gap by analyzing relevant literature to map research trajectories, assess current trends, identify emerging hotspots, and predict future directions. By offering a novel perspective, our findings may provide actionable insights for clinical decision-making and advance investigative efforts targeting diabetes and sarcopenia.

## 2 Materials and methods

### 2.1 Data acquisition and search strategy

The Web of Science Core Collection (WOSCC), provided by Clarivate Analytics, is a prestigious citation database that covers a wide range of disciplines, including natural sciences, social sciences, arts, and humanities. It is recognized as one of the most reliable and publisher-independent citation databases globally (19), and is frequently used for bibliometric research and scientific writing (20).

Data collection was restricted to articles indexed up to December 26, 2024. We performed a topic-based search in the WOSCC using the following combined query: TS = (“sarcopenia” OR “sarcopenic” OR “muscle depletion” OR “muscle wasting” OR “muscle atrophy”) AND TS = (“diabetes” OR “diabetes mellitus” OR “diabetic” OR “diabetic mellitus” OR “diabete” OR “Type 1 Diabetes Mellitus” OR “T1DM” OR “Type 2 Diabetes Mellitus” OR “T2DM” OR “aged diabetics” OR “senile diabetes” OR “geriatric diabetes”). The specific search terms and strategy are outlined in Supplementary Table S1 (see Supplementary materials). The search, data extraction, and downloading were conducted on the same day to ensure consistency and accuracy, minimizing discrepancies due to data updates. An initial search yielded 3,037 records. After setting the document type to “Article” and “Review Article,” limiting the language to “English,” and after deduplication and screening, 2,779 articles were retained. The literature search and screening processes were independently conducted by two authors, with any discrepancies resolved through consensus discussion to ensure methodological rigor.

### 2.2 Data filtering and export

On the retrieval date, plain text files containing all relevant metadata (e.g., title, document type, authors, abstract, keywords, full text, and references) for the 2,779 publications were downloaded from the WOSCC. Each file was renamed as “download_xxx.txt” for subsequent analysis. These files were then imported into CiteSpace, VOSviewer, and R software for filtering, followed by manual validation to remove irrelevant records. After this rigorous process, 2,773 articles were retained for further analysis. The data processing procedure was independently performed by two authors, with any discrepancies resolved through discussion. Supplementary Figure S1 depicts the process of selection.

### 2.3 Bibliometric and visualized analysis

Bibliometric analysis, which examines citation networks and co-citation patterns to reveal knowledge structures, developmental trajectories, and research hotspots within academic domains, has been widely applied across disciplines (21). This study utilized four analytical tools for data visualization and analysis: CiteSpace (v6.4.R1), VOSviewer (v1.6.20), R (v4.4.2, with the Bibliometrix package) and Microsoft Office Excel (2019). CiteSpace and VOSviewer, both Java-based bibliometric visualization tools (22), are widely employed in academic research. CiteSpace (23, 24) integrates clustering analysis and burst detection algorithms to identify seminal publications, research evolution, and emerging trends in scientific literature, with a specific focus on detecting abrupt conceptual shifts (25). VOSviewer enhances domain comprehension through intuitive network mapping. The Bibliometrix R package in R supports comprehensive bibliometric workflows, including data retrieval, processing, statistical analysis, and visualization, enabling systematic exploration of academic trends, collaboration networks, and research frontiers. By synergizing these tools, this study established a comprehensive and systematic bibliometric analysis framework.

In this study, Microsoft Office Excel was used to analyze publication trends over time. CiteSpace was conducted for author and institution profiling, journal co-citation and dual-map overlays analysis, co-citation, clustering, and burst detection of literature, as well as keyword co-occurrence, clustering, burst detection, and timeline map visualization. VOSviewer was employed for mapping country/region collaboration networks, while R software was mainly used to quantify statistical publication outputs. Detailed parameter configurations for each tool are provided in Supplementary Table S2.

## 3 Results

### 3.1 Global perspective

Following systematic literature retrieval, screening, and data cleaning, 2,773 articles were ultimately included in the analysis. Collectively, these studies in the field of diabetes and sarcopenia research were authored by 14,912 researchers affiliated with 2,178 institutions across 87 countries or regions, covering 937 diverse journals. From the database inception to the retrieval date, the total citation count for these publications reached 100,355, with an average of 36.19 citations per article (Supplementary Figure S2). The h-index, a key metric used to quantify scholarly output and impact, is defined as the maximum value “h” where a researcher has “h” publications, with each article cited at least “h” times (26). For this research domain, the h-index attained 141 during the study period (1962–2024), reflecting both substantial productivity and sustained academic influence. It is noteworthy that only 21 (0.76%) of these articles focused on sarcopenia and type 1 diabetes mellitus (T1DM), whereas the vast majority investigated sarcopenia and T2DM.

### 3.2 Analysis of disciplinary categories

According to the WOS disciplinary classification system, the 2,773 publications in the diabetes and sarcopenia research domain spanned 87 distinct disciplinary categories. To clarify the academic distribution patterns, Table 1 lists the top 10 disciplines ranked by publication volume and their respective contributions. “Endocrinology Metabolism” ranked first with 664 publications (23.95%), followed by “Geriatrics Gerontology” and “Nutrition Dietetics,” each contributing 373 publications (13.45%). Additionally, “Medicine General Internal” (251publications, 9.05%) and “Biochemistry Molecular Biology” (202 publications, 7.28%) also demonstrated significant scholarly output.

**Table 1:** Top 10 disciplines ranked by publication output.

Rank	WOS categories	Publications	Percentage
1	Endocrinology metabolism	664	23.95%
2	Geriatrics gerontology	373	13.45%
3	Nutrition dietetics	373	13.45%
4	Medicine general internal	251	9.05%
5	Biochemistry molecular biology	202	7.28%
6	Cell biology	141	5.08%
7	Medicine research experimental	140	5.05%
8	Pharmacology pharmacy	121	4.36%
9	Multidisciplinary sciences	118	4.26%
10	Physiology	111	4.00%

Notably, research in diabetes and sarcopenia exhibits a pronounced interdisciplinary nature, encompassing fields such as endocrinology, geriatrics, nutrition, biology, and clinical medicine. This reflects a trend of cross-disciplinary integration and collaboration. Such synergistic interactions have not only enhanced the understanding of pathological mechanisms, clinical interventions, and preventive strategies for diabetes and sarcopenia but also accelerated the development of this research field, providing multidimensional perspectives and broad platforms for future research.

### 3.3 Analysis of trends in publication outputs

**Figure 1:**
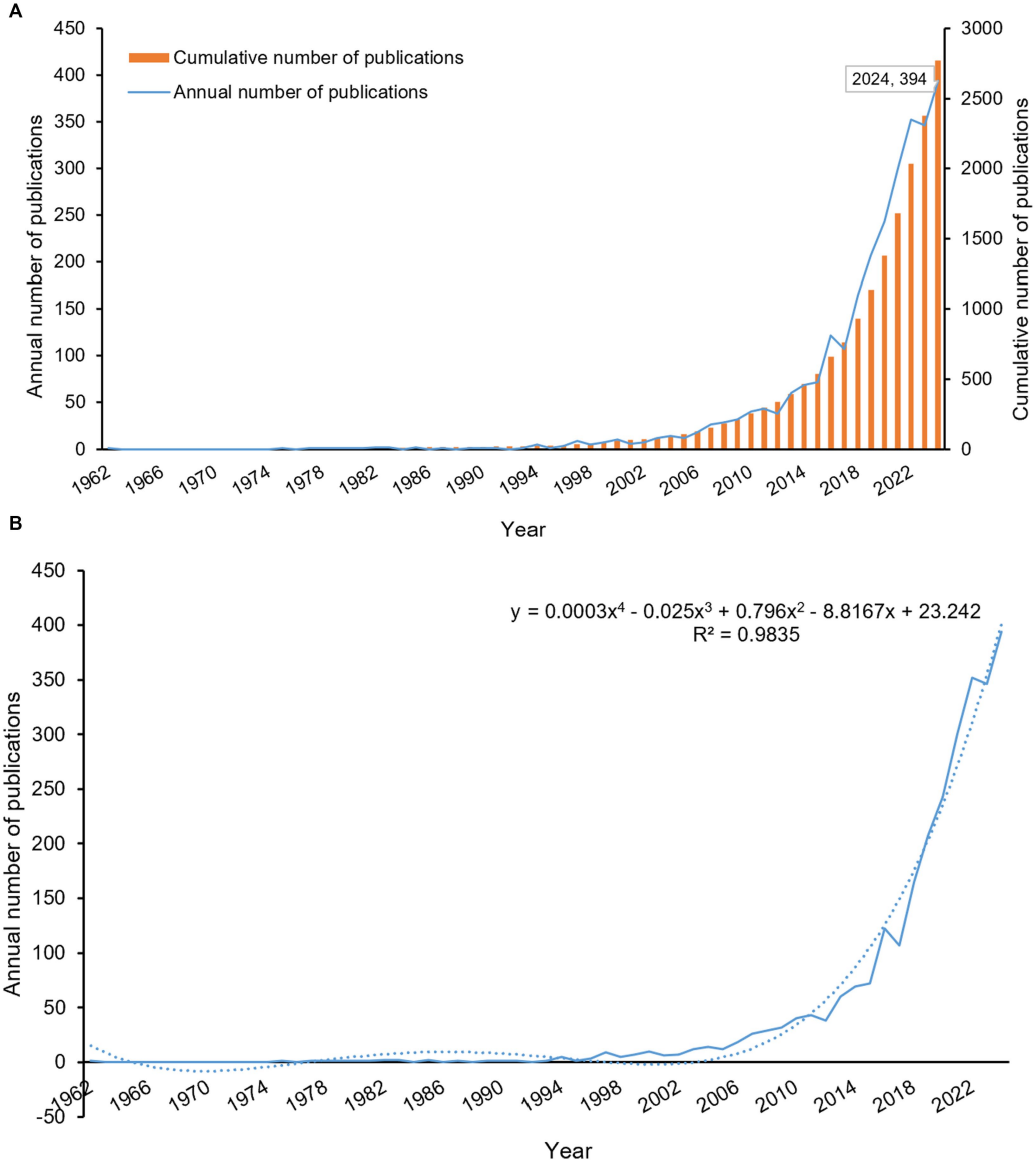
Analysis of publication trends from 1962 to 2024. **(A)** Annual publication volume and cumulative publication trends. **(B)** Annual publication trends with polynomial fitting curve. The polynomial fitting curve yielded the equation: *y = 0.0003x^4^–0.025x^3^ + 0.796x^2^–8.8167x + 23.242* (R^2^ = 0.9835), with x corresponding to the year and y indicating the yearly publication output.

A temporal analysis of 2,773 publications revealed the evolving research output in diabetes and sarcopenia. Figure 1A illustrates the annual publication trends, demonstrating a sustained upward trajectory from 1962 to December 26, 2024, with a peak of 394 publications in 2024 (note: 2024 data include records up to December 26). Polynomial regression was applied to further characterize this trend (Figure 1B), which yielded the equation *y = 0.0003x^4^–0.025x^3^ + 0.796x^2^–8.8167x + 23.242* (*R*^2^ = 0.9835), with x corresponding to the year and y indicating the yearly publication output. *R*^2^ values closer to 1 indicate a good fit. Publications exhibited a sustained annual growth rate of 10.12% (Supplementary Figure S2).

Although the term “sarcopenia” was first introduced by geriatrician Irwin Rosenberg in the late 1980s (27) and formally published in 1993 (28), early investigations into diabetes-associated muscle atrophy date back to 1962 (29). This early research documented asymmetric muscle wasting and proximal limb weakness in two of five patients with diabetic peripheral neuropathy, attributed to combined motor-sensory nerve dysfunction. Subsequent formalization of sarcopenia as a clinical entity, coupled with global population aging (30) and the rising prevalence of diabetes (31), has driven increasing scholarly attention to sarcopenia as an emerging comorbidity in diabetes. The interplay between diabetes and sarcopenia has now emerged as a research priority, with anticipated growth in both publication volume and investigator engagement in this field.

### 3.4 Analysis of countries/regions

**Figure 2:**
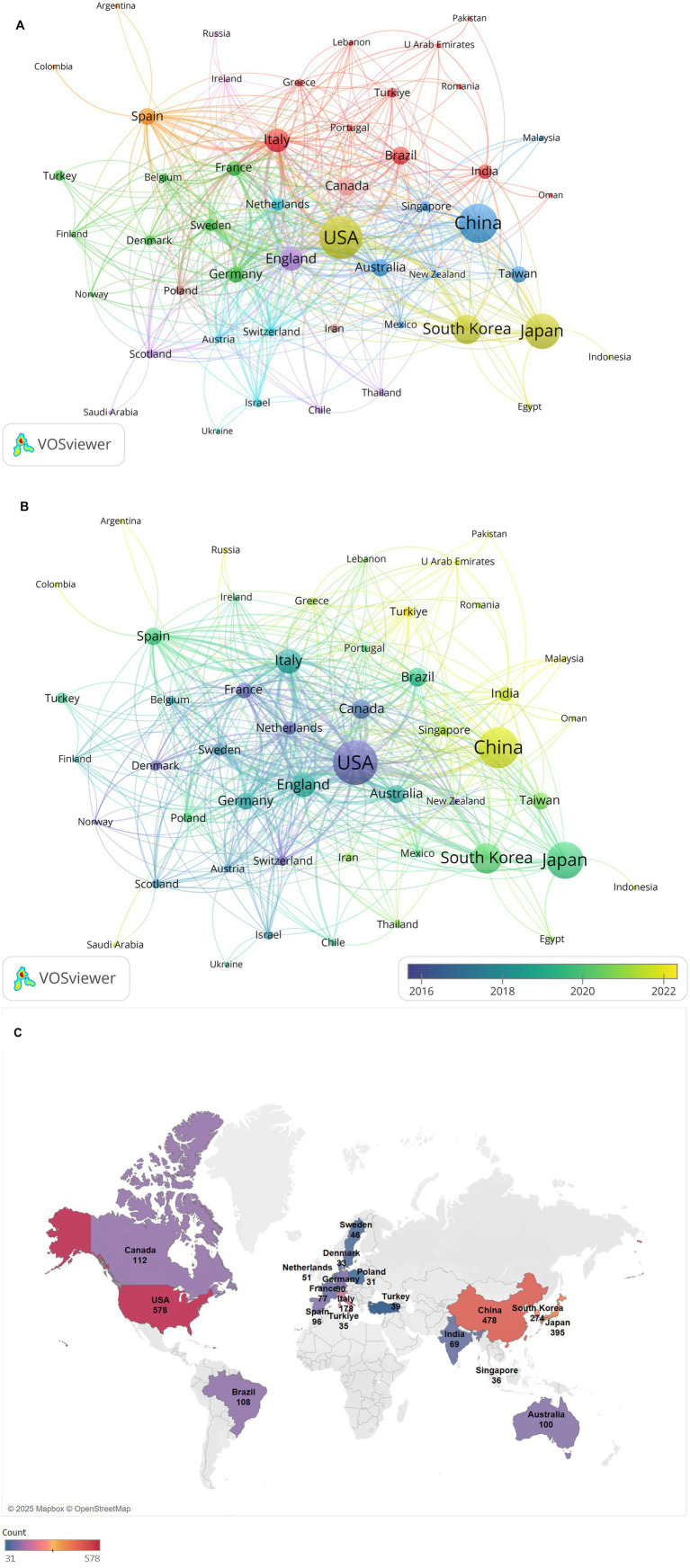
Analysis of countries/regions collaboration network. **(A)** Visualization of the countries/regions collaboration network. Nodes (circles) represent individual countries/regions, with node size proportional to their publication volume. Edges (lines) denote collaborative relationships between countries/regions, where edge thickness reflects the strength of collaboration. **(B)** Time-overlay visualization of the countries/regions collaboration network. Node color intensity corresponds to the average publication year (warmer hues indicate more recent contributions). **(C)** Geographical distribution map of different countries/regions.

Figure 2A presents a country collaboration network map generated using VOSviewer, illustrating global research collaborations in diabetes and sarcopenia. Publications in this field span 87 countries or regions worldwide. Table 2 lists the top 10 countries/regions by publication output, with the United States, China, Japan, South Korea, and Italy occupying the top five positions. The United States leads with the highest publication volume, totaling 578 papers, followed by China with 478 papers, and Japan with 395 papers. In terms of total citation frequency, the United States, Italy, the United Kingdom, Japan, and South Korea rank in the top five, with the United States having the highest total citation count at 43,305, followed by Italy with 13,238 and England with 10,183. The United States leads in both publication output and total citation count, underscoring its central role, with its research outcomes having broad global influence and recognition. Additionally, collaboration analysis reveals the United States holds the highest total link strength (379), with dense connections to China, Japan, Germany, and Italy, reflecting strong international partnerships. Although England ranks sixth in publication volume, its total link strength (238) reflects active global engagement.

**Table 2:** Top 10 countries or regions ranked by publication output.

Rank	Country/Region	Publications	Total citations	Total link strength
1	USA	578	43,305	379
2	China	478	8,018	109
3	Japan	395	9,242	70
4	South Korea	274	8,776	67
5	Italy	178	13,238	216
6	England	169	10,183	238
7	Canada	112	6,652	100
8	Brazil	108	2,637	69
9	Australia	100	4,290	119
10	Spain	96	3,166	123

“Total link strength” refers to the level of connection or collaboration between different countries or regions.

Figure 2B illustrates the collaboration network between countries or regions over time, reflecting the average publication years of research articles from high-output countries or regions. Early collaboration hubs (2016–2018) centered on the United States, Germany, and Canada, whereas recent research outputs (2020–2024) predominantly originate from Asian countries like China and India, signaling rising regional influence.

Furthermore, Figure 2C shows a geographical distribution map, demonstrates regional clustering of publications in North America, East Asia, and Europe. European nations exhibit strong intra-regional collaborations while maintaining close ties with the United States and China. This multifaceted nature of international collaboration not only expands the scope of academic partnerships but also fosters cross-cultural knowledge exchange and innovation.

### 3.5 Analysis of institutions and authors

A total of 2,178 institutions and 10,289 authors contributed to the research on diabetes and sarcopenia. To identify high-productivity and high-impact entities, Tables 3, 4 present the top 10 institutions and authors by publication volume, respectively, highlighting their academic contributions and influence. As shown in Table 3, Harvard University (United States) ranked first globally with 62 publications. Among the top 10 institutions, six were based in the United States, including Veterans Health Administration (46 publications), US Department of Veterans Affairs (46 publications), University of California System (44 publications), Harvard Medical School (43 publications), and University of Texas System (37 publications). This distribution underscores the dominant role of the United States in diabetes and sarcopenia research, consistent with its status as the leading country in terms of publication output. Additionally, Seoul National University (52 publications) and Yonsei University (49 publications) from South Korea demonstrated significant contributions, reflecting the growing research impact of Asian institutions in this field. Fukui Michiaki emerged as the most prolific author with 42 publications and 750 total citations, demonstrating exceptional contributions to the field (Table 4). Hamaguchi Masahide (38 publications, 645 citations) and Hashimoto Yoshitaka (37 publications, 735 citations) followed closely, with their high citation counts indicating the substantial academic impact of their research.

**Table 3:** Top 10 institutions ranked by publication output.

Rank	Institution	Country	Publications	Percentage
1	Harvard University	USA	62	2.24%
2	Seoul National University (SNU)	Korea	52	1.88%
3	Yonsei University	Korea	49	1.77%
4	Veterans Health Administration	USA	46	1.66%
5	Kyoto Prefectural University of Medicine	Japan	46	1.66%
6	US Department of Veterans Affairs	USA	46	1.66%
7	University of London	England	45	1.62%
8	University of California System	USA	44	1.59%
9	Harard Medical School	USA	43	1.55%
10	University of Texas System	USA	37	1.33%

**Table 4:** Top 10 authors ranked by publication output.

Rank	Author	Publications	Percentage	Total citations
1	Fukui, Michiaki	42	1.51%	750
2	Hamaguchi, Masahide	38	1.37%	645
3	Hashimoto, Yoshitaka	37	1.33%	735
4	Okamura, Takuro	31	1.11%	450
5	Yamazaki, Masahiro	28	1.01%	613
6	Ushigome, Emi	25	0.90%	465
7	Nakanishi, Naoko	21	0.08%	242
8	Okada, Hiroshi	21	0.08%	256
9	Senmaru, Takafumi	20	0.07%	237
10	Majima, Saori	18	0.06%	232

### 3.6 Analysis of journals

**Figure 3:**
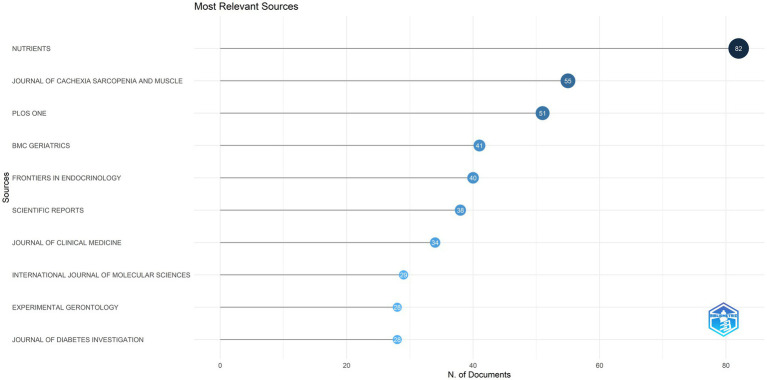
The top 10 journals in terms of publication output. This figure displays the top 10 journals in diabetes and sarcopenia research, which together published 426 articles, accounting for 15.36% of the total publications.

Figure 3 presents the top 10 journals in diabetes and sarcopenia research, collectively publishing 426 articles (15.36% of total publications). *Nutrients* ranked first with 82 publications, followed by the *Journal of Cachexia Sarcopenia and Muscle* (55 publications) and *PloS One* (51 publications), indicating a relatively dispersed publication distribution.

**Figure 4:**
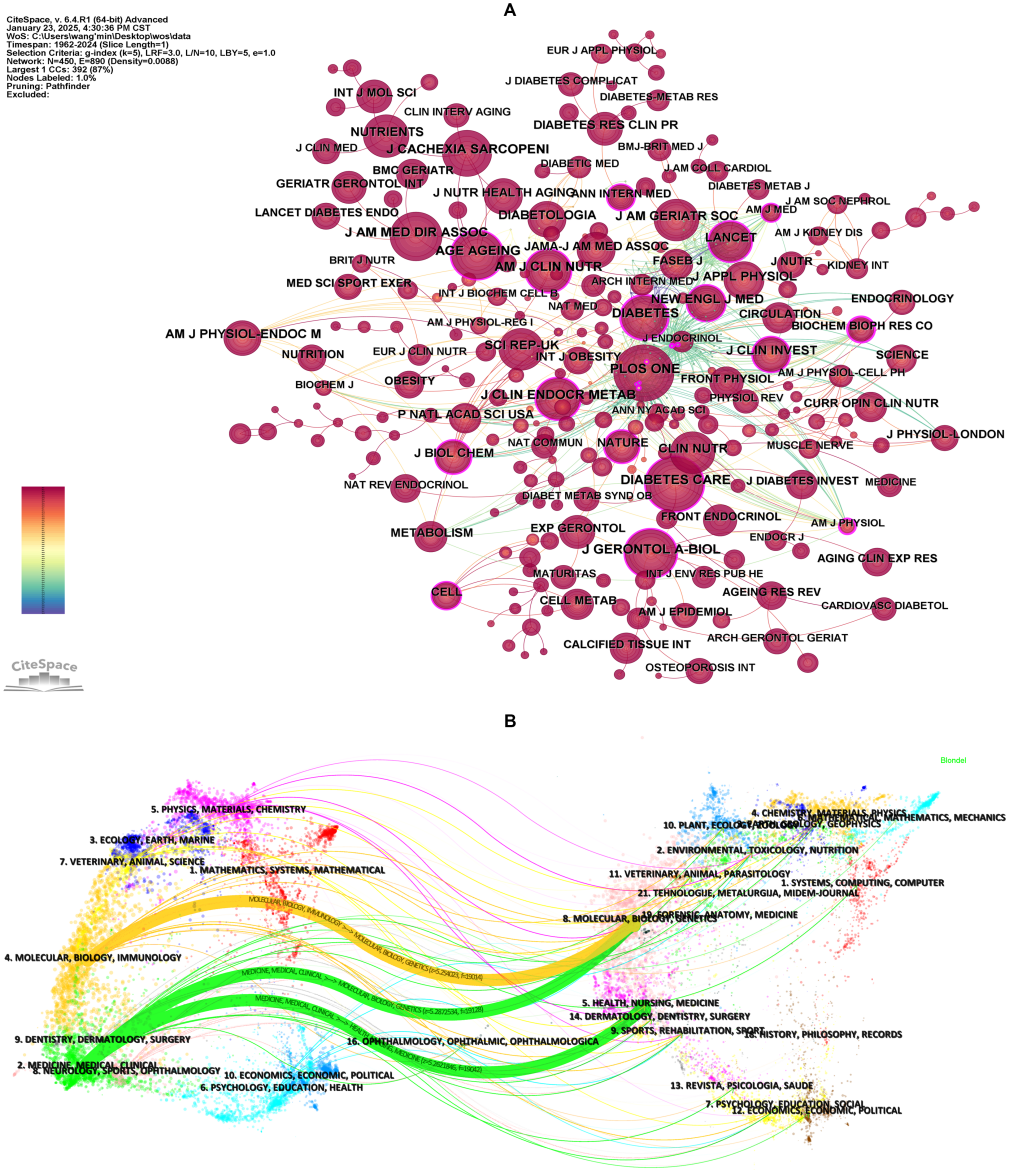
Analysis of co-cited journals. **(A)** Visualization of journal co-citation network. Nodes represent journals, with node size proportional to their co-citation frequency (larger nodes indicate higher co-citation counts). Node centrality reflects the structural influence of journals within the network, where higher centrality values denote greater connectivity and pivotal roles in knowledge dissemination. **(B)** The dual-map overlay of journals. The left side represents citing journals, while the right side represents cited journals. The curved lines between them represent citation links, indicating the citation paths, with the thickness of the lines proportional to citation frequency.

Journal co-citation, defined as the frequency with which two or more journals are cited together in reference lists, reveals inter-journal relationships (32). It serves as a key metric for assessing academic reputation and disciplinary influence. Figure 4A and Table 5 display the co-citation network of journals and the top 10 most co-cited journals in this field. *PloS One* (1,316 co-citations) ranked highest, followed by *Diabetes Care* (1,295 co-citations) and *Journals of Gerontology Series A-Biological Sciences and Medical Sciences* (1,098 co-citations). Notably, most of the top co-cited journals exhibit high impact factors (IFs) (e.g., *Diabetes Care*: IF = 14.80, *Journal of Cachexia Sarcopenia and Muscle*: IF = 9.40) and belong to the JCR Q1 quartile, indicating their authority in this field.

**Table 5:** Top 10 co-cited journals in terms of number of co-citations.

Rank	Co-cited journal	Co-citation frequency	Centrality	IF (2024)	JCR quartile (2024)
1	PloS One	1,316	0.00	2.90	Q1
2	Diabetes Care	1,295	0.17	14.80	Q1
3	Journals of Gerontology Series A-Biological Sciences and Medical Sciences	1,098	0.11	4.30	Q1
4	Age and Ageing	1,003	0.12	6.00	Q1
5	Journal of the American Medical Directors Association	984	0.03	4.20	Q2
6	Diabetes	976	0.27	6.20	Q1
7	Journal of Cachexia Sarcopenia and Muscle	887	0.05	9.40	Q1
8	Journal of Clinical Endocrinology & Metabolism	858	0.14	5.0	Q1
9	American Journal of Clinical Nutrition	857	0.10	6.50	Q1
10	Journal of the American Geriatrics Society	847	0.01	4.30	Q1

IF: Impact Factor.

Moreover, co-citation patterns reflect interdisciplinary collaboration. Journals in diabetes, geriatrics, and clinical nutrition dominated the rankings, aligning with the cross-disciplinary nature of diabetes and sarcopenia research. Centrality analysis revealed *Diabetes* (centrality = 0.27) as a critical connector in the collaboration network, whereas journals like *the Journal of the American Geriatrics Society* showed lower centrality despite high impact factors, suggesting narrower disciplinary focus. A notable overlap exists between the top 10 journals by publication volume and co-citation frequency (e.g., *PloS One*), highlighting journals that balance productivity with scholarly impact.

A dual-map overlay is a visual representation that maps the citation relationships between citing and cited journals (33). This overlay provides a comprehensive view of how research in a specific field is interconnected with other disciplines, highlighting the interdisciplinary flow of knowledge. Using CiteSpace, we generated a dual-map overlay for diabetes and sarcopenia research (Figure 4B), with journal citation links merged based on z-scores. Supplementary Table S3 lists the names of the citing and cited regions for the three primary citation links. Within the cited journal clusters, major disciplines include Molecular, Biology, Genetics, Health, Nursing and Medicine. The three core citation trajectories originate from these fields and have evolved into cutting-edge research areas such as Molecular, Biology, Immunology, Medicine, Medical, and Clinical. In addition, disciplines like Physics, Materials, Chemistry, Veterinary, Animal and Science, may emerge as new frontiers for future research.

### 3.7 Analysis of co-cited references

**Figure 5:**
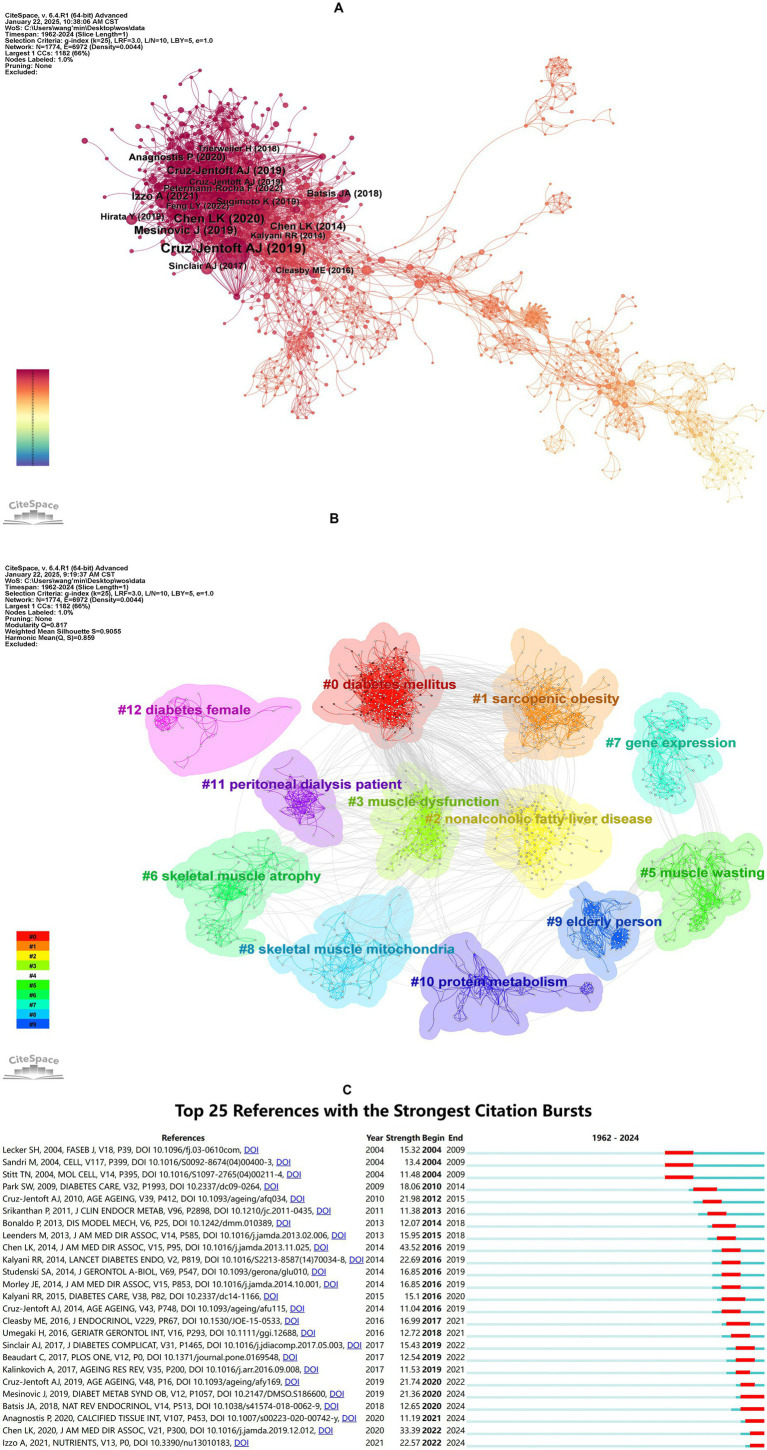
Analysis of co-cited reference. **(A)** Co-citation network visualization of references. Nodes represent individual references, with node size proportional to their co-citation frequency (larger nodes indicate higher co-citation counts). Edges denote co-citation relationships between references, where edge thickness reflects co-citation strength (thicker edges indicate stronger associations between paired references). **(B)** Clustering network analysis of references. **(C)** Top 25 references with the strongest citation bursts. “Strength” quantifies the intensity of citation bursts, while “Begin” and “End” demarcate the burst duration (indicated by red segments). Longer red spans correspond to sustained periods of heightened citation activity.

Co-citation references analysis, which identifies relationships between two references cited together by a third document, helps uncover core literature, research hotspots, and thematic connections within a field, providing critical insights into its knowledge structure and developmental trajectory (34). Figure 5A illustrates the co-citation network, while Table 6 summarizes the top 10 co-cited references, all published between 2014 and 2021, with co-citation frequencies exceeding 50. The most frequently co-cited reference was the article “Sarcopenia: Revised European Consensus on Definition and Diagnosis” by Cruz-Jentoft et al. (35), published in *Age and Aging* in 2019. This study updated the European Working Group on Sarcopenia in Older People (EWGSOP) definition of sarcopenia, emphasizing low muscle strength as a core feature and poor physical performance as a marker of severe sarcopenia. It also provided clear diagnostic thresholds and clinical algorithms to improve diagnostic consistency, promote early detection and intervention, and advocated for further research to reduce the burden of sarcopenia on patients and healthcare systems.

**Table 6:** The top ten highly cited papers on the co-citation references.

Rank	Title	First author	Journal	Year	Co-citation frequency
1	Sarcopenia:revised European consensus on definition and diagnosis	Cruz-Jentoft AJ	Age and Aging	2019	389
2	Asian Working Group for Sarcopenia: 2019 Consensus Update on Sarcopenia Diagnosis and Treatment	Chen LK	Journal of the American Medical Directors Association	2020	243
3	Sarcopenia and type 2 diabetes mellitus: a bidirectional relationship	Mesinovic J	Diabetes Metabolic Syndrome and Obesity	2019	157
4	Sarcopenia	Cruz-Jentoft AJ	Lancet	2019	134
5	A Narrative Review on Sarcopenia in Type 2 Diabetes Mellitus: Prevalence and Associated Factors	Izzo A	Nutrients	2021	114
6	Sarcopenia in Asia: Consensus Report of the Asian Working Group for Sarcopenia	Chen LK	Journal of the American Medical Directors Association	2014	82
7	Type 2 Diabetes Mellitus is Associated with Increased Risk of Sarcopenia: A Systematic Review and Meta-analysis	Anagnostis P	Calcified Tissue International	2020	76
8	Sarcopenic obesity in older adults: aetiology, epidemiology and treatment strategies	Batsis JA	Nature Reviews Endocrinology	2018	64
9	Hyperglycemia in non-obese patients with type 2 diabetes is associated with low muscle mass: The Multicenter Study for Clarifying Evidence for Sarcopenia in Patients with Diabetes Mellitus	Sugimoto K	Journal of Diabetes Investigation	2019	55
10	Frailty and sarcopenia - newly emerging and high impact complications of diabetes	Sinclair AJ	Journal of Diabetes and Its Complications	2017	53

Notably, the review “Sarcopenia and Type 2 Diabetes Mellitus: A Bidirectional Relationship” is ranked third among the top co-cited references. This review establishes a reciprocal causal link between T2DM and sarcopenia mediated by insulin resistance, chronic inflammation, and mitochondrial dysfunction. The authors emphasize that sarcopenia exacerbates T2DM progression through impaired glucose disposal and ectopic fat accumulation, while T2DM accelerates muscle decline via oxidative stress and advanced glycation end-products (36). The high co-citation frequency of this paper underscores the growing recognition of the bidirectional interplay between sarcopenia and diabetes in the literature.

To correct for citation age bias, we have included a citation density analysis in the Supplementary materials (see Supplementary Table S4).

Building on the co-citation network, clustering and burst detection analyses were conducted. Figure 5B presents a clustered network with a modularity *Q* value of 0.8170 (>0.3000) and a mean silhouette *S* value of 0.9055 (>0.7000), indicating a significant and reliable clustering structure. The network comprises 12 clusters, each with a corresponding label. The clusters can be divided into three main themes: diabetes and its metabolic complications, muscle pathology and dysfunction, and special populations and clinical research, as detailed in Supplementary Table S5.

Figure 5C displays the top 25 references with the strongest burst detection. The reference with the highest burst strength (Strength = 43.52) was “Sarcopenia in Asia: Consensus Report of the Asian Working Group for Sarcopenia,” which outlined the diagnostic consensus developed by the Asian Working Group for Sarcopenia (AWGS). This consensus emphasized the combined assessment of muscle mass, strength, and physical performance and proposed diagnostic thresholds tailored to Asian populations. Additionally, five references have exhibited bursts extending to 2024, focusing on the bidirectional relationship between diabetes and sarcopenia, the impact of diabetes on muscle health, and the importance of early identification and intervention. These studies have highlight the significance of lifestyle interventions, nutritional supplementation, and personalized treatments in improving the health of diabetic patients.

In summary, early definitions and diagnostic criteria for sarcopenia varied due to regional, ethnic, and methodological differences. However, efforts by global sarcopenia working groups have led to increasing standardization in diagnosis and assessment. This progress provides a clear framework for research on the relationship between diabetes and sarcopenia, advancing precision medicine and optimizing clinical intervention strategies.

### 3.8 Analysis of keywords

**Figure 6:**
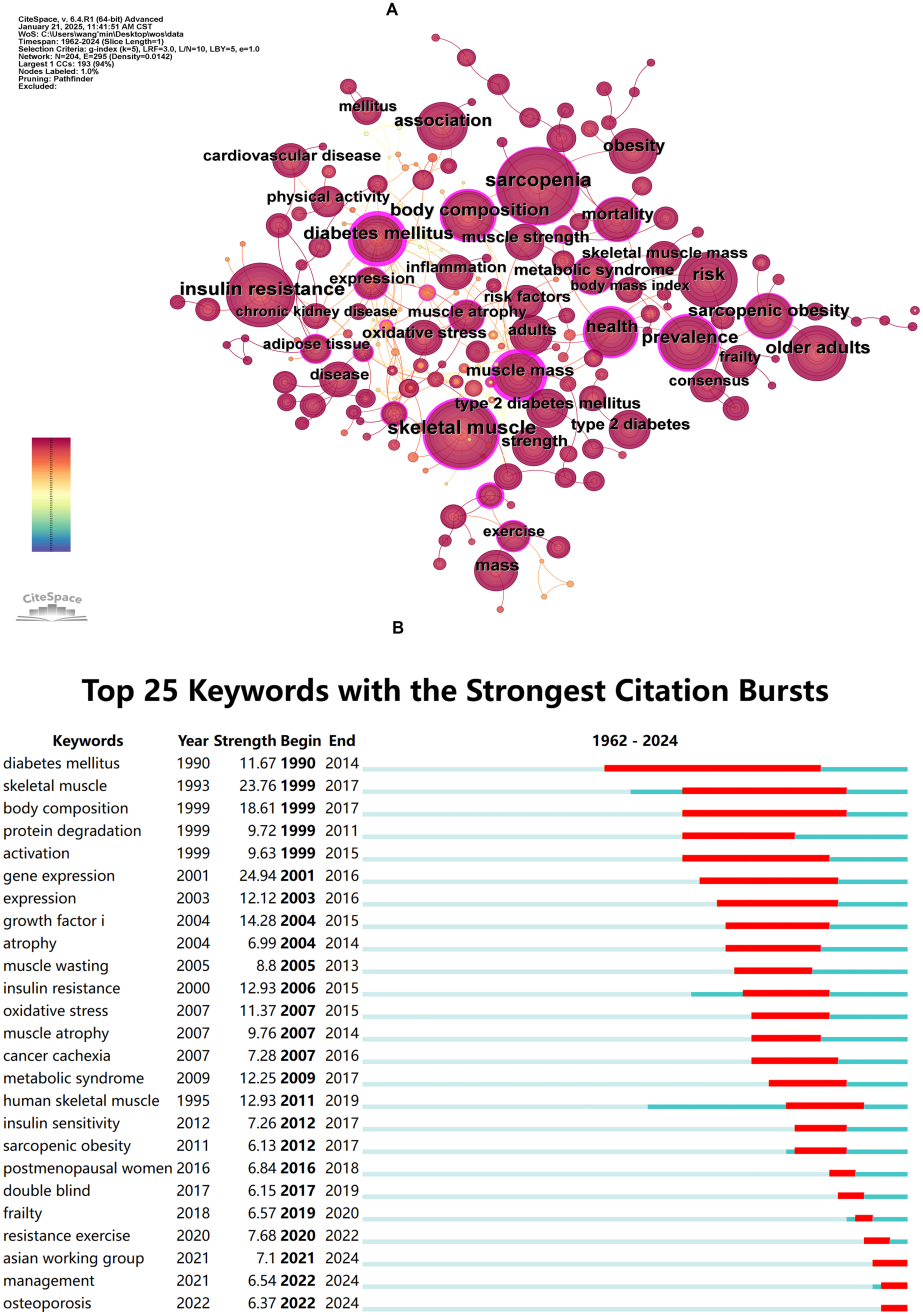
Co-occurrence and burst analysis of keywords. **(A)** Keyword co-occurrence network map. **(B)** Top 25 keywords with the strongest citation bursts.

Keyword co-occurrence analysis was performed to identify the interrelationships among keywords in diabetes and sarcopenia research. The co-occurrence network is shown in Figure 6A and the top 20 high-frequency keywords are listed in Table 7. “Sarcopenia” (621 occurrences) “skeletal muscle” (586 occurrences) “insulin resistance” (535 occurrences) “risk” (342 occurrences) and “body composition” (332 occurrences) ranked highest in frequency. Notably “muscle mass” exhibited the highest centrality (0.70) despite its lower frequency. Half of the top 20 keywords demonstrated centrality values exceeding 0.1 indicating their pivotal bridging roles in connecting diverse research themes.

**Table 7:** The top 10 most frequent keywords.

Rank	Keyword	Occurrence	Centrality
1	Sarcopenia	621	0.16
2	Skeletal muscle	586	0.32
3	Insulin resistance	535	0.07
4	Risk	342	0.04
5	Body composition	332	0.20
6	Older adults	322	0.00
7	Diabetes mellitus	319	0.71
8	Prevalence	313	0.24
9	Health	294	0.40
10	Association	279	0.02
11	Obesity	256	0.09
12	Sarcopenic obesity	253	0.13
13	Mortality	233	0.13
14	Muscle mass	222	0.71
15	Mass	209	0.02
16	Metabolic syndrome	191	0.12
17	Strength	190	0.00
18	Muscle strength	184	0.09
19	Type 2 diabetes	175	0.00
20	Adults	173	0.05

Burst detection analysis (Figure 6B) revealed emerging trends, with “gene expression” (Strength = 24.94), “skeletal muscle” (Strength = 23.76), and “body composition” (Strength = 23.76) ranking as the top three keywords with the highest burst strength. The longest burst duration was observed for “diabetes mellitus” persisting from 1990 to 2014, while “Asian working group,” “management,” and “osteoporosis” have remained active to the present.

**Figure 7:**
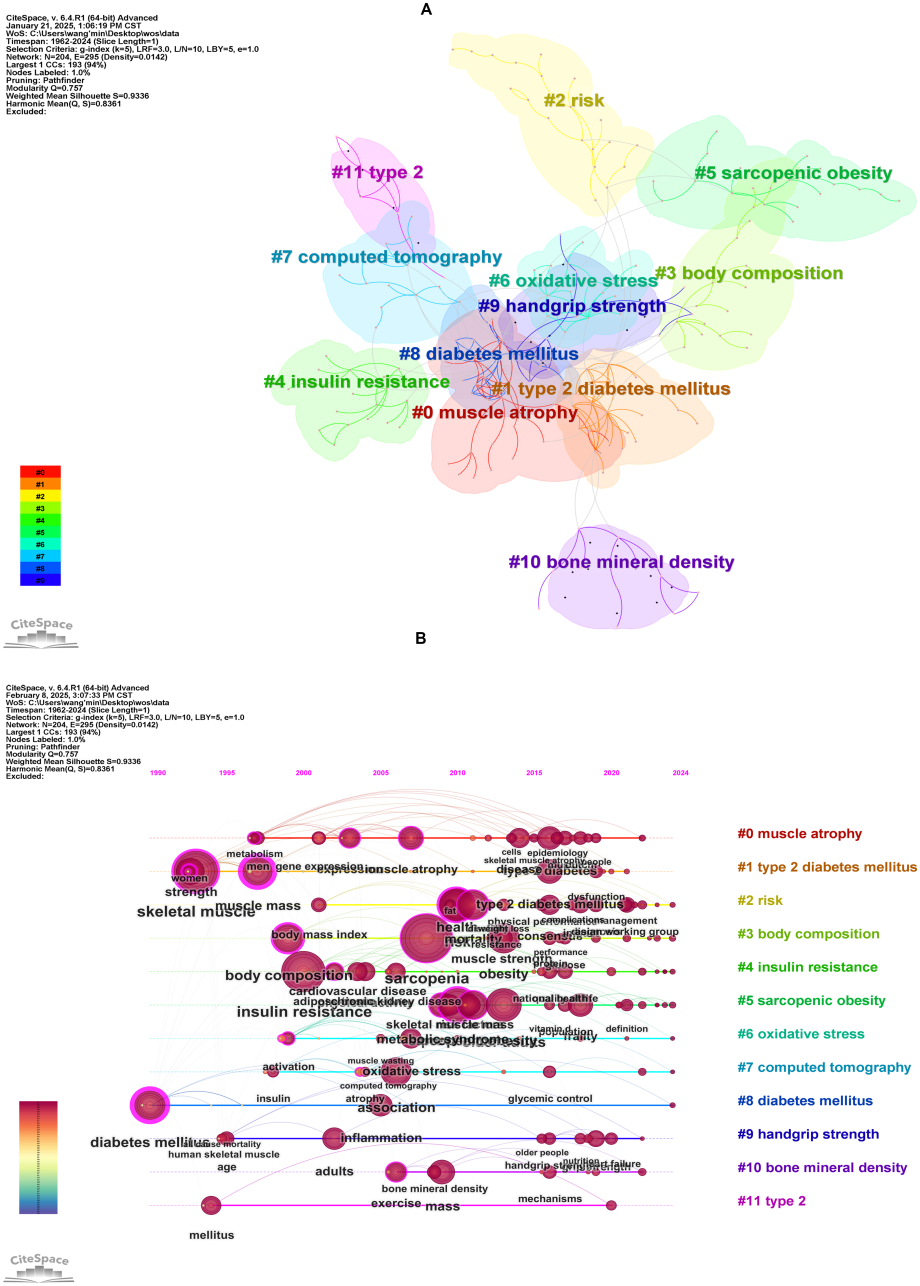
Cluster analysis of keywords. **(A)** Keywords clustering visualization map. **(B)** The timeline visualization map based on cluster analysis.

Keywords clustering analysis (Figure 7A) generated 12 clusters with a modularity *Q* value of 0.7570 (>0.3000) and a mean silhouette *S* value of 0.9336 (>0.7000) confirming robust network structure. The most active cluster was #0 muscle atrophy which included 23 keywords such as “muscle atrophy” “type 2 diabetes” “glucocorticoids” and “pyroptosis.” The second most active cluster was #1 type 2 diabetes mellitus which included 21 keywords such as “type 2 diabetes mellitus” “muscle mass” “skeletal muscle” “strength” and “metformin.” These 12 clusters can be grouped into four primary research areas: shared pathological mechanisms muscle function assessment and imaging-based assessment and diagnosis.

Timeline analysis (Figure 7B) illustrated the shifting focus of research themes. Early studies (prior to the 1990s) centered on “diabetes mellitus” and “insulin resistance” primarily focused on metabolic mechanisms of diabetes. Between 2000 and 2010, “muscle atrophy” and “body composition” emerged as key topics, reflecting increased attention to diabetes-induced musculoskeletal deterioration. From 2010 to 2020, keywords like “sarcopenic obesity,” “oxidative stress,” and “handgrip strength” signified a deeper exploration of the interplay between muscle dysfunction, ectopic fat deposition, and metabolic disorders in diabetic patients. Since 2020, cross-disciplinary terms such as “metabolic syndrome,” “frailty,” and “cardiovascular disease” have dominated, making a shift from studying single-organ pathophysiology to multi-system joint assessments. This shift emphasizes the synergistic impact of diabetes and sarcopenia on overall health outcomes, such as cardiovascular events and functional decline. Notably, Persistent emphasis on “insulin resistance” and “oxidative stress” reaffirms their role as key pathological links, while terms like “exercise” underscore the importance of lifestyle interventions in the integrated management of these conditions. Emerging evidence supports the therapeutic efficacy of physical exercise in managing sarcopenia among diabetic patients, with resistance training leading to significant improvements in muscle mass and strength, while aerobic exercise enhances skeletal muscle function (37). Furthermore, a recent bibliometric study by Zang et al. (38), which focused on exercise in sarcopenia, identified three primary research themes: resistance exercise alone, multimodal exercise regimens, and exercise combined with nutritional supplementation. Their analysis further revealed that resistance training remains the dominant research focus, particularly in its effects on grip strength. Building upon these established findings, our results demonstrate that exercise-based strategies play an important role in the clinical management of diabetic sarcopenia.

The overall research trajectory demonstrates an evolution from mechanistic exploration to clinical integration, ultimately advancing toward multidimensional precision interventions.

## 4 Discussion

### 4.1 General overview

This study is the first to provide a bibliometric overview of global research on diabetes and sarcopenia. Data analysis shows a steady increase in annual publications from database inception to December 26, 2024, with a notable acceleration after 2014 (Figure 1A). This acceleration coincides with the adoption of standardized sarcopenia diagnostic criteria (e.g., AWGS updates) and growing recognition of its clinical significance. Over time, research emphasis has shifted from single disease studies to bidirectional interactions. Currently, sarcopenia is recognized as a complication of diabetes, and diabetes itself is considered an independent risk factor for sarcopenia progression. When both conditions coexist, patients face worse outcomes, including higher fall rates, metabolic dysregulation, and increased all-cause mortality. The peak publication count in 2024 underscores the field’s continued rapid expansion.

Globally, 2,178 institutions across 87 countries/regions contributed 2,773 publications in 87 disciplines. The geographical distribution map showed North America, East Asia, and Europe as core research regions for research output. The United States dominated with the highest publication volume (578 articles, 20.84%) and total citation frequency (43,305), followed by China (478 articles, 17.24%). The United States and China together account for approximately one-third of all publications, reflecting substantial national investment in diabetes and muscle research and aligning with their high prevalence of diabetes-sarcopenia comorbidity in aging populations. European nations demonstrated a closely knit collaborative network, while maintaining strong ties with the United States and China. This international collaboration underscores the importance of shared scientific knowledge and resources in tackling global health challenges associated with sarcopenia.

Institutional analysis revealed that among the top 10 institutions by publication volume, six were based in the United States, with Harvard University (62 papers) in the lead. Fukui Michiaki, as the most prolific author with a total of 42 research articles, demonstrated remarkable consistency and productivity in his scholarly contributions, which merits attention in the analysis of authorship.

With respect to journal distribution, the journal *Nutrients* published the most relevant articles, followed by the *Journal of Cachexia, Sarcopenia and Muscle*, and *PloS One*. The leading role of *Nutrients* indicates a translational focus on nutritional and metabolic interventions. This aligns with current clinical practice guidelines recommending protein supplementation and anti-inflammatory diets alongside pharmacotherapy in diabetic sarcopenia management. Among the most cited journals, *PloS One* (1,316 citations) ranks the highest, followed by *Diabetes Care* (1,295 citations) and *The Journals of Gerontology: Series A, Biological Sciences and Medical Sciences* (1,098 citations). The interdisciplinary characteristic of this field is significant, encompassing endocrinology, geriatrics medicine, nutrition, biology, clinical medicine, and other fields. This multidimensional approach reflects the necessity of addressing the disease from different perspectives.

### 4.2 Research foci and frontiers

Based on the analysis of co-citation references and keywords, the hot topics and frontiers in the field of diabetes and sarcopenia research are summarized. However, it must be emphasized that most research has focused on the relationship between T2DM and sarcopenia, with relatively few studies examining sarcopenia in T1DM. This research imbalance may be attributed to the relatively low prevalence of T1DM (accounting for approximately 5–10% of the cases of diabetes) and its complex pathophysiology, including autoimmune-mediated *β*-cell destruction, which has contributed to the underappreciation of distinct muscle metabolic abnormalities in T1DM.

#### 4.2.1 Exploration of shared pathological mechanisms

Insulin resistance, chronic low-grade inflammation, oxidative stress, mitochondrial dysfunction, and adipose-muscle crosstalk represent key shared pathological mechanisms underlying both diabetes and sarcopenia (14). As a key insulin-targeted organ, skeletal muscle plays a crucial role in glucose uptake and metabolism, which are vital for maintaining systemic glucose homeostasis. Insulin resistance impairs the phosphoinositide 3-kinase (PI3K)/protein kinase B (Akt)/mechanistic target of rapamycin signaling pathway (e.g., aberrant insulin receptor substrate 1 phosphorylation), suppressing muscle protein synthesis while activating the ubiquitin-proteasome system and autophagy-lysosomal pathways, promoting muscle atrophy (39). Hyperglycemia-induced accumulation of advanced glycation end products exacerbates mitochondrial oxidative phosphorylation dysfunction via the receptor for advanced glycation end products signaling, leading to excessive generation of reactive oxygen species (ROS). ROS directly damage muscle cells by oxidizing DNA, proteins, and lipids, while simultaneously activating the nuclear factor kappa B (NF-κB) and c-Jun N-terminal kinase pathways, upregulating pro-inflammatory cytokines (e.g., tumor necrosis factor-alpha [TNF-*α*] and interleukin [IL]-6), further inhibiting PI3K/Akt signaling and enhancing proteolysis. Furthermore, ROS impairs muscle satellite cell self-renewal and differentiation through the p38 mitogen-activated protein kinase pathway, thereby hindering the regenerative capacity of muscles. Chronic low-grade inflammation serves as a central hub in both conditions: the senescence-associated secretory phenotype drives the secretion of adipose tissue–derived inflammatory mediators (e.g., IL-6, TNF-α, C-reactive protein), exacerbating insulin resistance via Janus kinase/signal transducer and activator of transcription and inhibitor of kappa B kinase/ NF-κB signaling, while activating muscle-specific E3 ubiquitin ligases (e.g., muscle RING finger 1 and muscle atrophy F-box) to accelerate protein degradation. Adipose-muscle crosstalk also involves dysregulated myokine-mediated modulation of lipid metabolism (e.g., irisin and myostatin) (36).

Future research should focus on the networked interplay of these mechanisms, particularly emerging areas such as mitochondrial-endoplasmic reticulum interactions and the gut microbiota-immune axis, to elucidate the comorbid basis of diabetes and sarcopenia and identify shared therapeutic targets.

#### 4.2.2 Research on specific subtypes: sarcopenic obesity (SO)

Clinicians frequently associate sarcopenia with weight loss, often overlooking its potential coexistence with obesity. In recent years, SO, defined as the co-existence of excess adiposity and low muscle mass/function (40), has gained growing attention as a distinct clinical subtype. This operational definition has been widely adopted in clinical practice. Compared to sarcopenia alone, SO poses greater health risks, including increased disability rates, mortality risks, and higher healthcare expenditures.

Emerging studies have investigated the pathophysiological links between SO and diabetes, involving mechanisms such as malnutrition, insulin resistance, chronic low-grade inflammation, and hormonal alterations (41). From a bibliometric perspective, current research predominantly focuses on the epidemiological characteristics of SO and the identification of associated risk factors and prognostic indicators. However, significant gaps remain in the development of preventive strategies, early diagnostic approaches, and therapeutic interventions for SO patients (42).

A study by Park et al. (43), based on the Korean Guro Diabetes Project involving 515 newly diagnosed T2DM patients, evaluated the predictive efficacy of waist circumference, waist-to-hip ratio, waist-to-height ratio, and weight-adjusted waist circumference index (WWI) for SO. Results demonstrated that WWI, as a novel anthropometric parameter, exhibited superior predictive performance for SO and cardiometabolic risks in T2DM patients, particularly in older populations (43). Another retrospective cohort study by Chuan et al. (44) analyzed 386 elderly patients with T2DM by using the AWGS criteria in combination with five obesity indices, including body mass index, body fat percentage (BF%), and visceral fat area, to assess the prevalence of SO and its association with adverse health outcomes. The study found that the prevalence of SO varied with different obesity indices, with BF%-defined SO being significantly associated with all-cause mortality, fragility fractures, and cardiovascular events. This suggests that BF% may be an optimal diagnostic indicator for adverse outcomes related to SO in elderly diabetic patients (44). Additionally, a cross-sectional study indicated that irisin may serve as a predictive biomarker for SO in T2DM patients (45).

#### 4.2.3 Application of imaging techniques to assess muscle function in patients with diabetes

In diabetic patients, alongside conventional muscle function assessments such as grip strength, calf circumference, daily step count, and the Short Physical Performance Battery, advanced imaging technologies—including dual-energy X-ray absorptiometry (DXA), computed tomography (CT), and magnetic resonance imaging (MRI) (46)—are pivotal for both qualitative and quantitative muscle evaluation, demonstrating significant clinical utility. As advocated by the EWGSOP2 criteria (35), the assessment of muscle function in sarcopenia also relies on imaging modalities such as DXA, CT, and MRI.

Shen et al. (47) pioneered a method to estimate whole-body skeletal muscle and adipose tissue using a single abdominal CT cross-sectional scan, building upon prior CT-based body composition analyses. CT imaging allows for precise visualization of muscle mass and fat infiltration, enabling quantitative measurements of muscle cross-sectional area and density to objectively assess muscle atrophy and fatty replacement. The application of CT-derived cutoff values is critical in this context. A retrospective study of 4,470 Korean adults established sex-specific reference values for chest skeletal muscle area (measured via pectoralis, intercostal, paraspinal, serratus anterior, and latissimus dorsi muscles) using thoracic CT scans from a healthy young reference cohort (aged 19–39 years). These standardized values enhance sex-specific sarcopenia diagnosis and chest muscle quantification in Asian populations (48). Beyond conventional CT, studies have explored contrast-enhanced CT (49) and peripheral quantitative CT (50) for skeletal muscle evaluation. Despite its efficacy, CT’s clinical adoption is limited by high radiation exposure, cost, and procedural complexity (9).

MRI, as a non-invasive, high-resolution imaging modality, is considered the gold standard for body composition analysis. It enables precise quantification of muscle mass and fat distribution, offering advantages in early sarcopenia diagnosis and disease progression monitoring. However, its high operational costs and prolonged scanning times restrict its widespread clinical use (51). Diffusion tensor imaging (DTI), an MRI-based technique, has proven sensitive to microstructural muscle changes. A study using DTI revealed that reduced white matter integrity in the left anterior thalamic radiation and right inferior fronto-occipital fasciculus of elderly diabetic patients correlates significantly with sarcopenia (52), suggesting potential links between central nervous system alterations and diabetes-associated sarcopenia. Furthermore, foot MRI studies in diabetic populations have identified pronounced muscle atrophy (sarcopenic changes), particularly in patients with diabetic neuropathy. The severity of atrophy correlates with clinical neuropathy scores and diabetic foot complications (53).

DXA and bioelectrical impedance analysis (BIA) are widely used for body composition assessment, particularly in muscle mass quantification. DXA uses two low-dose X-ray beams to differentiate bone mineral density, fat mass, and lean mass (including muscle and non-fat tissues). BIA estimates body fat and lean mass by measuring electrical impedance through surface electrodes, exploiting differences in tissue conductivity (higher in hydrated muscle compared to fat). However, BIA accuracy is influenced by hydration status, dietary intake, and physical activity, necessitating complementary methods for robust results.

Collectively, each imaging modality presents distinct advantages and limitations. CT and MRI provide high-precision quantitative data but face barriers due to radiation exposure, cost, and complexity. Conversely, DXA and BIA offer cost-effective and accessible alternatives but lack the specificity of dedicated muscle composition analyses (e.g., MRI or CT). To address the growing need for early sarcopenia detection in diabetes, researchers should prioritize the development of sensitive imaging biomarkers, including shear-wave elastography and grayscale ultrasound, alongside novel molecular markers to improve diagnostic and screening accuracy.

## 5 Limitations

Our study has some limitations. First, as a bibliometric analysis, our approach inherently emphasizes quantitative trends (e.g., publication volume, citation counts) over qualitative assessments of study validity or clinical impact. While citation metrics reflect academic attention, they do not necessarily correlate with methodological rigor or real-world healthcare relevance. For instance, highly cited articles might emphasize mechanistic hypotheses rather than clinically actionable interventions. Second, the data were obtained only from WOSCC, potentially omitting relevant studies indexed in other databases such as PubMed, Scopus, and Embase. Nonetheless, WOSCC remains a leading source for bibliometric investigations, offering comprehensive citation tracking essential for analyzing research outputs, trends, and academic influence. Third, the exclusion of non-English publications may introduce selection bias, potentially overlooking contributions from non-English language studies. Future work should expand data sources to include multilingual literature and incorporate longitudinal datasets, which would enhance global representativeness and allow for more comprehensive bibliometric analyses.

## 6 Conclusion

This study used bibliometric methods to comprehensively evaluate the scientific landscape of diabetes and sarcopenia research. Research in these fields has progressed from exploring pathological mechanisms to the development of diagnostic imaging techniques and therapeutic approaches, reflecting a gradual shift in the theoretical framework from basic pathology toward clinical applications. Building on this groundwork, we also sought to identify existing research gaps and shortcomings, proposing new directions for further exploration.

The link between diabetes and sarcopenia in individuals with T1DM requires deeper investigation. Future research could capitalize on trends in interdisciplinary integration and precision medicine, such as: (1) integrated single-cell and spatial multi-omics to map muscle microenvironment changes and identify molecular targets; (2) AI-driven digital biomarkers and personalized rehabilitation using wearable sensors and machine-learning models for early detection and customized interventions; and (3) community-based ultrasound AI screening for non-invasive, scalable sarcopenia detection.

In summary, future research that integrates multidisciplinary approaches will enhance health outcomes for patients with diabetes and sarcopenia.

## Data Availability

The data analyzed in this study is subject to the following licenses/restrictions: The data for this study were sourced from the publicly accessible Web of Science database (https://www.webofscience.com/wos/). Data are available upon reasonable request. Requests to access these datasets should be directed to liyuntao@njmu.edu.cn.
