# Supplementary figures and images for: Bibliometric mapping of diabetes mellitus and sarcopenia research: hotspots and emerging trends
Source: Front Med (Lausanne). 2025 May 27;12:1586308. doi: 10.3389/fmed.2025.1586308 (PMC12148892; doi:10.3389/fmed.2025.1586308)

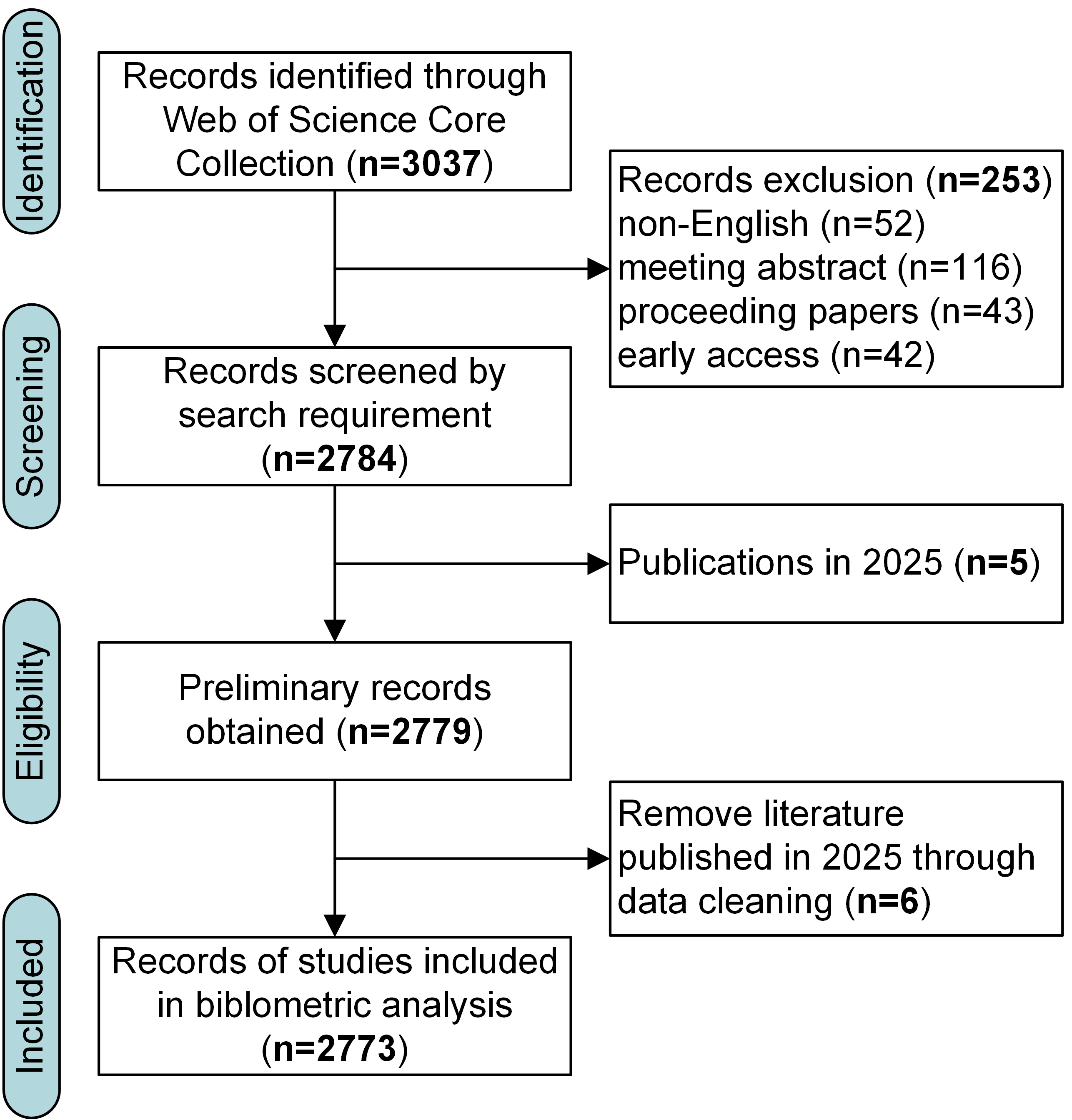

Supplement: Supplementary file 1 [file Data_Sheet_1.zip › Supplementary Figures/Figure S1 .tif]

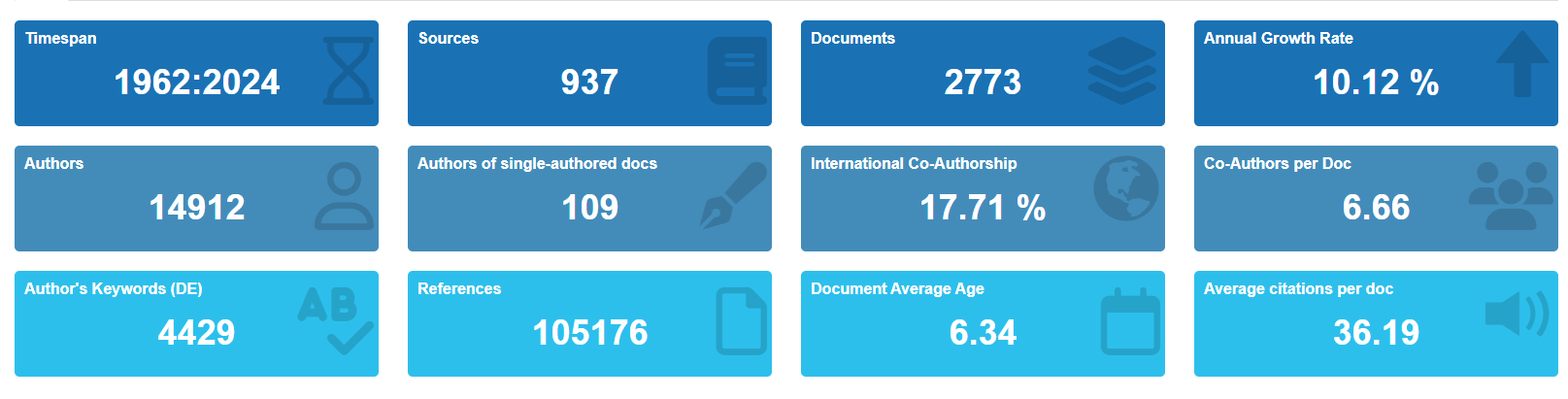

Supplement: Supplementary file 1 [file Data_Sheet_1.zip › Supplementary Figures/Figure S2 .tif]
