# Supplementary material for: Bibliometric mapping of diabetes mellitus and sarcopenia research: hotspots and emerging trends
Source: Front Med (Lausanne). 2025 May 27;12:1586308. doi: 10.3389/fmed.2025.1586308 (PMC12148892; doi:10.3389/fmed.2025.1586308)
Supplement: Supplementary file 2 [file Table_1.docx]

**Supplementary Materials**

1. **Supplementary Figures**

Figure S1 Flowchart of literature screening

Figure S2 Statistical analysis of publications using R software

1. **Supplementary Tables**

Table S1 Search strategy in Web of Science Core Collection

| #1. TS=(“sarcopenia” OR “sarcopenic” OR “muscle depletion” OR “muscle wasting” OR “muscle atrophy”)  #2. TS=(“diabetes” OR “diabetes mellitus” OR “diabetic” OR “diabetic mellitus” OR “diabete” OR “Type 1 Diabetes Mellitus” OR “T1DM” OR “Type 2 Diabetes Mellitus” OR “T2DM” OR “aged diabetics” OR “senile diabetes” OR “geriatric diabetes”)  #3. #1 AND #2 |
| --- |
| Note:  The search was conducted on December 26, 2024, using citation indexes including Science Citation Index Expanded(SCI-EXPANDED), Social Sciences Citation Index(SSCI), Arts & Humanities Citation Index(AHCI), Conference Proceedings Citation Index-Science(CPCI-S), Conference Proceedings Citation Index-Social Science(CPCI-SSH), Emerging Sources Citation Index(ESCI), Current Chemical Reactions(CCR-EXPANDED),Index Chemicus(IC). |

Table S2 Settings of software parameters

| CiteSpace v6.4.R1 | Time slicing: 1962-2024 |
| --- | --- |
|  | Years per slice: 1 |
|  | Links strength: Cosine |
|  | Selection criteria: g-index |
|  | Pruning: Pathfinder |
|  | Clustering algorithm: Log-Likelihood Ratio(LLR) |
|  | Node display method: Tree Ring History |
| VOSviewer v1.6.20 | Minimum number of documents of a country: 5;  Of the 87 countries, 49 meet the thresholds. |
| R 4.4.2 | None required |
| Microsoft Office Excel 2019 | None required |

Table S3 Key citation trends

| Citing region | Cited Region |
| --- | --- |
| Molecular, Biology, Immunology | Molecular, Biology, Genetics |
| Medicine, Medical, Clinical | Molecular, Biology, Genetics |
| Medicine, Medical, Clinical | Health, Mursing, Medicine |

Note: Journals in molecular, biology and immunology frequently cite literature from molecular, biology and genetics, while publications in medicine, clinical, and medical commonly draw references from health, nursing, medicine, molecular, genetics and biology.

Table S4 Statistical analysis of clustering topics in literature co-citation

| ID | Cluster theme | Cluster labels |
| --- | --- | --- |
| 1 | Diabetes and its metabolic-related complications | #0 diabetes mellitus, #1 sarcopenic obesity, #2 nonalcoholic fatty liver disease, #12 diabetes female |
| 2 | Muscle pathology and dysfunction | #3 muscle dysfunction, #5 muscle wasting, #6 skeletal muscle atrophy, #7 gene expression, #8 skeletal muscle mitochondria, #10 protein metabolism |
| 3 | Special populations and clinical research | #9 elderly person, #11 peritoneal dialysis patient |
